# Supplementary material for: Photonic chip-based soliton frequency combs covering the biological imaging window
Source: Nat Commun. 2018 Mar 20;9:1146. doi: 10.1038/s41467-018-03471-x (PMC5861103; doi:10.1038/s41467-018-03471-x)
Supplement: Supplementary file 1 — Supplementary information(PDF 10279 kb) [file 41467_2018_3471_MOESM1_ESM.pdf]

Supplementary Information - Photonic chip-based soliton frequency combs covering the biological imaging window

Maxim Karpov, Martin H. P. Pfeiffer, Junqiu Liu, Anton Lukashchuk, Tobias J. Kippenberg
École Polytechnique Fédérale de Lausanne (EPFL), CH-1015 Lausanne, Switzerland
(Dated: February 15, 2018)

Supplementary Note 1

Dispersion engineering of Si_3N_4-microresonators at 1064 nm

In order to experimentally demonstrate the ability to engineer the dispersion of silicon nitride microresonators at around 1-µm wavelength, one needs to show a consistent trend in the measured dispersion, when the geometry of the devices is changed. However, due to large free spectral range of our devices (~ 1 THz) and small dispersion terms (e.g. $D_2/2\pi \sim 20 - 30$ MHz), one has to measure resonance frequencies within a broad range of several hundreds of nanometers in order to estimate the dispersion with an acceptable level of precision [1, 2].

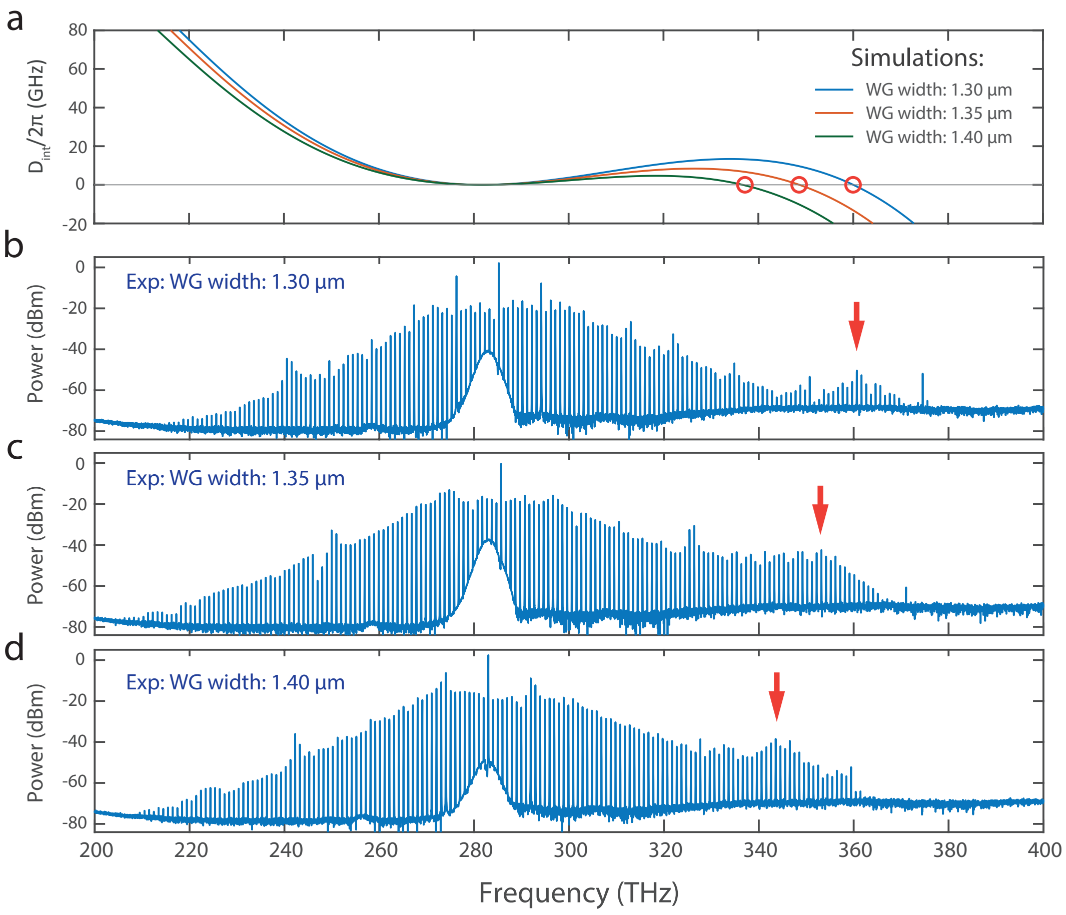

Supplementary Figure 1. **Dispersion engineering of Si_3N_4-microresonators at 1064 nm.** (a) FEM-based simulations of integrated dispersion ($D_{\mathrm{int}}/2\pi$) in 1-THz silicon nitride microring resonator with waveguide height of 0.74 µm, various waveguide widths of 1.30, 1.35, 1.40 µm, and sidewall angle of 77°. Red circles show spectral position of the phase-matching points ($D_{\mathrm{int}}/2\pi = 0$); (b)-(d) Experimental optical spectra of the fabricated 1-THz Si_3N_4 microresonators with the height of 0.74 µm and waveguide widths of 1.30, 1.35 and 1.40 µm (by design). The positions of the short-wavelength phase-matching regions are indicated with red arrows.

Instead of doing this, one can measure the spectral locations of the phase-matching-induced enhancements of comb lines in the noisy comb states, which correspond to $D_{\mathrm{int}}/2\pi = 0$ and provide a rough estimation for the position of soliton dispersive wave for a given geometry [3]. The positions of such phase-matched regions depend strongly on

dispersion parameters and are easily tracked in experiment, allowing for convenient comparison to simulations. It is important, however, to highlight here that the generation of solitons is not needed for these measurements, and may even give improper results, because the exact spectral position of the dispersive in a soliton state is slightly shifted from the phase-matched region where $D_{\mathrm{int}}/2\pi = 0$ [4].

We measured optical spectra of noisy Kerr comb states (i.e. operating in regime of chaotic modulation instability) in fabricated devices with a different geometry (height of 0.74 µm and widths of 1.30, 1.35, 1.40 µm) and traced the positions of the short-wavelength phase-matching region (see Supplementary Figure 1 (b-d)). The positions are marked with red arrows, and demonstrate an increasing trend from 343 THz to 360 THz as the waveguide widths decreases.

We have also simulated the dispersion profile for the same set of the resonator waveguide geometries with height of 0.74 µm, widths of 1.30, 1.35 and 1.40 µm and sidewall angle of 77°. The positions of phase-matching points (marked with circles in Fig.1(a)) show the same trend as in fabricated samples, and coincide well with the experimentally obtained phase-matching regions.

Supplementary Note 2

Dissipative Kerr solitons in hybridized modes

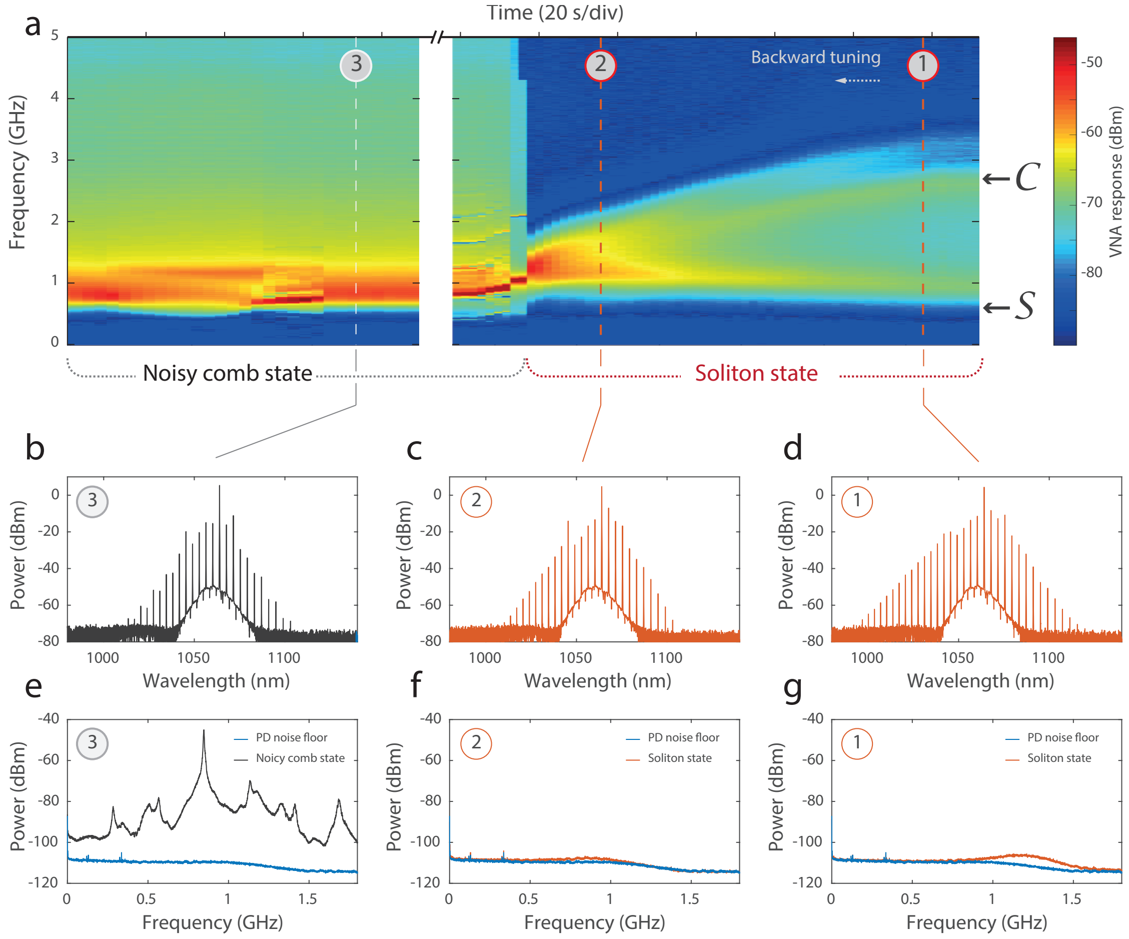

Supplementary Figure 2. **Characterization of dissipative Kerr soliton states in hybridized modes.** (a) Evolution of the response measurements of the state obtained by pumping the hybridized mode at 1067 nm (resonance (ii) in Fig.4(b) of the main text). The state evolves from the right to the left, following the backward tuning sweep of the pump laser (from longer to the shorter wavelengths). Initial positions of $\mathcal{C}-$ and $\mathcal{S}-$resonances are indicated with $\mathcal{C}$ and $\mathcal{S}$. (b) - (d) Optical spectra obtained at different stages (1-3) of the backward tuning shown in (a). (e) - (h) Intensity noise of the total comb power obtained at different stages (1-3) of the backward tuning shown in (a).

In this section we closely investigated one of the dissipative Kerr soliton (DKS) states, obtained in hybridized modes in the last section of the manuscript. We have used the same device as in the main manuscript and generated the state pumping the resonance at 1067 nm (resonance (ii) in the Fig.4 of the main manuscript). In order to unambiguously demonstrate the formation of DKS, we have generated the considered state, and implemented a slow backward pump tuning when the pump laser was sweeping towards shorter wavelengths with a speed < 0.5 GHz/s. At the same time we monitored the state using pump-phase-modulation-based system response [5], as well as the measured optical spectrum of the state and its intensity noise. Supplementary Figure 2(a) shows the evolution of the system response from the initial state (on the right) to the final state (on the left). Response measurement of the initial state reveals an existence of two response peaks, which we attribute to the cavity ($\mathcal{C}$-) and soliton ($\mathcal{S}$-) contributions. While tuning the state backward [5], we can see how the response evolves: the two peaks are getting closer to each other indicating the reducing of the pump detuning. The spectrum of the state maintains a smooth envelope with moderate line-to-line variation except a small modal crossing at around 1050 nm. The low intensity noise also stays almost unchanged reaching the photodiode noise floor. After $\mathcal{C}$- and $\mathcal{S}$-resonances are merged, the system suddenly returns to the noisy comb state, which is indicated by broadband intensity noise (Fig.2(e)) and a structured optical spectrum (Fig.2(b)). These measurements are qualitatively similar to the evolution of response measurements of the bright soliton states in Si_3N_4 and MgF_2 platforms at 1550 nm, when the system switches from the DKS to a comb state [5].

Supplementary Note 3

Mode interactions in Si_3N_4-microresonators

Mode interactions that happens in our microresonator and lead to dispersion alterations differ from the mode interactions often observed in integrated waveguides due to the phase-matching between different transverse mode families (matching of effective refractive indices)[6]. In our case, the mode interaction process, which induces dispersion deviation used for DKS is caused by Rayleigh scattering from surface and volume inhomogeneities as well as the bus-resonator coupler. In order to demonstrate this fact we simulated effective refractive indices for fundamental and two high-order modes in our waveguide (see Supplementary figure 3). It can be seen, that the fundamental mode families (TE_{00}, TM_{00}) do not cross over the full simulated frequency range, and the main process enabling the mode interaction here (if the modes are frequency-degenerate) is scattering. We note that this mode hybridization process is different from the phase-matching-based mode hybridization used in the work by Lee *et al.* [7], which is inherent to the geometry.

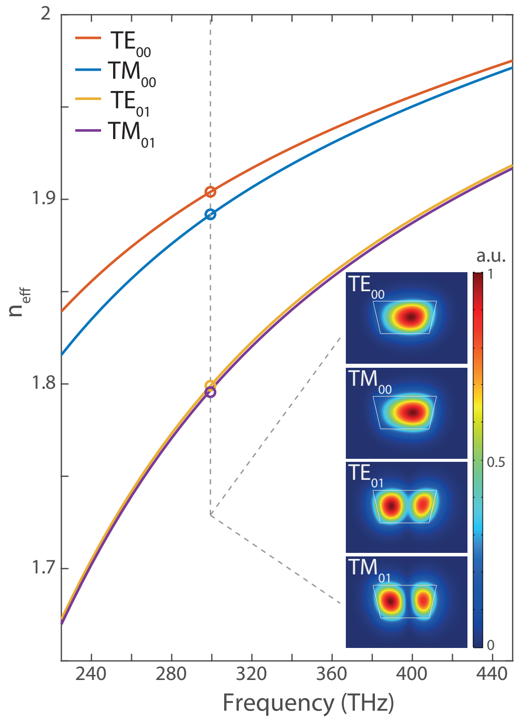

Supplementary Figure 3. **Mode families of silica-cladded Si_3N_4-microresonator.** Effective refractive indices of different transverse mode families in silica-cladded silicon nitride waveguide with waveguide width of 1.45 µm, height of 0.74 µm and sidewall angle of 77°. Insets show the normalized field distribution of four modes: TE_{00}, TM_{00}, TE_{01} and TM_{01} obtained for the optical frequencies marked with the dashed line.

Supplementary References

[1] Del'Haye, P., Arcizet, O., Gorodetsky, M. L., Holzwarth, R. & Kippenberg, T. J. Frequency comb assisted diode laser spectroscopy for measurement of microcavity dispersion. *Nat. Photon.* **3**, 529–533 (2009).

[2] Liu, J. *et al.* Frequency-comb-assisted broadband precision spectroscopy with cascaded diode lasers. *Opt. Lett.* **41**, 3134–3137 (2016).

[3] Brasch, V. *et al.* Photonic chip–based optical frequency comb using soliton Cherenkov radiation. *Science* **351**, 357–360 (2016).

[4] Cherenkov, A. V., Lobanov, V. E. & Gorodetsky, M. L. Dissipative kerr solitons and cherenkov radiation in optical microresonators with third-order dispersion. *Phys. Rev. A* **95**, 033810 (2017).

[5] Guo, H. *et al.* Universal dynamics and deterministic switching of

dissipative kerr solitons in optical microresonators. *Nat. Physics* **13**, 94–102 (2017).

[6] Dai, D. & Zhang, M. Mode hybridization and conversion in silicon-on-insulator nanowires with angled sidewalls. *Optics express* **23**, 32452–32464 (2015).

[7] Lee, S. H. *et al.* Towards visible soliton microcomb generation. *Nature Communications* **8**, 1295 (2017).
